# Molecular Modeling Study of a Receptor–Orthosteric Ligand–Allosteric Modulator Signaling Complex

**DOI:** 10.1021/acschemneuro.2c00554

**Published:** 2023-01-24

**Authors:** Chen Jiang, Xibing He, Yuanqiang Wang, Chih-Jung Chen, Yasmin Othman, Yixuan Hao, Jiayi Yuan, Xiang-Qun Xie, Zhiwei Feng

**Affiliations:** † Department of Pharmaceutical Sciences and Computational Chemical Genomics Screening Center, Pharmacometrics & System Pharmacology (PSP) PharmacoAnalytics, School of Pharmacy; National Center of Excellence for Computational Drug Abuse Research; Drug Discovery Institute; Departments of Computational Biology and Structural Biology, School of Medicine, University of Pittsburgh, Pittsburgh, Pennsylvania15261, United States; ‡ School of Pharmacy and Bioengineering, Chongqing University of Technology, Chongqing400054, China; § Chongqing Key Laboratory of Medicinal Chemistry and Molecular Pharmacology, Chongqing400054, China; ∥ Chongqing Key Laboratory of Target Based Drug Screening and Effect Evaluation, Chongqing400054, China

**Keywords:** MD simulation, orthosteric binding pocket, allosteric modulator, β2 adrenoceptor

## Abstract

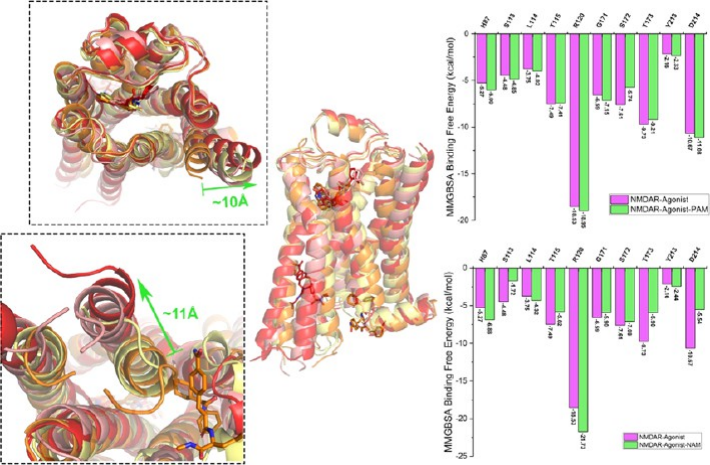

Allosteric modulators (AMs) are considered as a perpetual hotspot in research for their higher selectivity and various effects on orthosteric ligands (OL). They are classified in terms of their functionalities as positive, negative, or silent allosteric modulators (PAM, NAM, or SAM, respectively). In the present work, 11 pairs of three-dimensional (3D) structures of receptor–orthosteric ligand and receptor–orthosteric ligand–allosteric modulator complexes have been collected for the studies, including three different systems: GPCR, enzyme, and ion channel. Molecular dynamics (MD) simulations are applied to quantify the dynamic interactions in both the orthosteric and allosteric binding pockets and the structural fluctuation of the involved proteins. Our results showed that MD simulations of moderately large molecules or peptides undergo insignificant changes compared to crystal structure results. Furthermore, we also studied the conformational changes of receptors that bound with PAM and NAM, as well as the different allosteric binding sites in a receptor. There should be no preference for the position of the allosteric binding pocket after comparing the allosteric binding pockets of these three systems. Finally, we aligned four distinct β2 adrenoceptor structures and three *N*-methyl-d-aspartate receptor (NMDAR) structures to investigate conformational changes. In the β2 adrenoceptor systems, the aligned results revealed that transmembrane (TM) helices 1, 5, and 6 gradually increased outward movement from an enhanced inactive state to an improved active state. TM6 endured the most significant conformational changes (around 11 Å). For NMDAR, the bottom section of NMDAR’s ligand-binding domain (LBD) experienced an upward and outward shift during the gradually activating process. In conclusion, our research provides insight into receptor–orthosteric ligand–allosteric modulator studies and the design and development of allosteric modulator drugs using MD simulation.

## 1 Introduction

Pharmacological ligands are classified between orthosteric and allosteric ligands depending on how they bind to a receptor. Orthosteric ligands bind to the same binding site on the receptor as the natural endogenous agonist (neurotransmitter or hormone), whereas allosteric modulators (AMs) bind to a site(s) that will not be considered as an orthosteric pocket.^1^ An orthosteric and allosteric ligand can then bind to the same receptor simultaneously, forming a ternary complex, in which they affect each other’s binding affinity.^2^ Therefore, the efficacy of an orthosteric agonist inducing the functional response may be influenced by the binding of the allosteric modulator.^2^ Allosteric ligands have various advantages over orthosteric ligands in which they show higher selectivity among subtypes of a particular receptor family. Moreover, rather than activating receptors on their own, pure allosteric modulators instead adjust the activity of the proteins, and the effects of endogenous ligands finely while maintaining physiological signaling patterns in time and space.^3^

Exogenous chemicals are often small molecules that bind to a region on a receptor that is remote from the native orthosteric site and are commonly thought of as AMs in the context of pharmaceutical development.^4^ The reported AMs aid in providing knowledge of these compounds’ structural and physicochemical properties and may further provide the principles and justification for allosteric medicine development.^5^

AMs can be divided into three categories based on their functions: positive allosteric modulators (PAMs), negative allosteric modulators (NAMs), and silent allosteric modulators (SAMs, or neutral allosteric ligands). PAMs enhance the activation of orthosteric agonists by increasing their affinity or efficacy.^6^ Conversely, NAMs diminish the affinity or efficacy of the receptor’s agonists by reducing protein activation or stabilizing the inactive state of a receptor triggered by antagonists. SAMs do not affect the activity of orthosteric ligands, but they can occupy allosteric binding sites, preventing PAMs and NAMs from binding and acting allosterically.^7^

Focusing on the receptor–orthosteric ligand–allosteric modulator complex, our lab previously analyzed the X-ray crystal structures and cryo-EM structures of 11 pairs of complexes downloaded from the Protein Data Bank (https://www.rcsb.org/),^8^ including GPCRs, enzymes, ion channels, and transcription factors by our novel algorithm toolset, Molecular Complex Characterizing System (MCCS).^9^ The results indicated that AMs do not significantly impact the conformation of residues involved in the binding of orthosteric ligands and AMs,^10^ which allow us to confidently conduct virtual screening with either known or reliable allosteric pockets for further design and development of AM drug candidates. To further validate this conclusion and explore the allosteric binding pattern and the conformational changes during the state change, we conducted molecular dynamics (MD) simulations to investigate the stability and dynamics of the complexes. Specifically, the X-ray crystal and cryo-EM structures were recorded from a moment of the complex, which can be considered as the static state of the complex. Performing MD simulations allowed us to sample the dynamic conformations and predict the stable state of the complex in a dynamic process.^11^ MCCS calculations, which consist of rigid-scoring and rigid-docking functions, can be applied to analyze the energy contributions of the ligand–interacting residues.^12^ However, both receptors and ligands have flexible structures instead of rigid ones in nature. Therefore, MD simulations were conducted to simulate the dynamics of proteins and ligands within a complex.

In this study, we ran MD simulations on three systems containing different receptors: a G-protein-coupled receptor (GPCR), an enzyme, and an ion channel (details are shown in Table 1). GPCRs are seven transmembrane-spanning proteins that make up more than 1% of the human genome and over 30% of all therapeutic drug targets on the market.^13^ They are the most prominent family of membrane proteins, mediating the major responses to hormones and neurotransmitters as well as vision, olfaction, and taste.^14^ Here, we choose β2 adrenoceptor as the typical receptor of GPCR. In the rhodopsin-like family of GPCRs, the β2 adrenoceptor not only was the first GPCR to be cloned^15^ but also has been widely studied using a variety of biophysical and structural approaches.^16^ Thus, there are adequate crystal structure resources to perform comparisons and analysis.

**Table 1:** Details of All Complexes Collected in This Study

PDB ID	details
4LDE	β2 adrenoceptor + Agonist (BI167107)
6N48	β2 adrenoceptor + Agonist (BI167107) + PAM
2RH1	β2 adrenoceptor + Antagonist (Carazolol)
5X7D	β2 adrenoceptor + Antagonist (Carazolol) + NAM
2HYY	Tyrosine-protein kinase ABL1 + Inhibitor (Imatinib)
3PYY	Tyrosine-protein kinase ABL1 + Inhibitor (Imatinib) + PAM
2G2H	Tyrosine-protein kinase ABL1 + Inhibitor (PD166326)
1OPK	Tyrosine-protein kinase ABL1 + Inhibitor (PD166326) + NAM
5H8F	NMDAR + Agonist (Glutamic acid, Glycine)
5H8H	NMDAR + Agonist (Glutamic acid, Glycine) + PAM
5H8N	NMDAR + Agonist (Glutamic acid, Glycine) + NAM

Enzymes are catalysts that essentially increase the rate of all chemical reactions within cells.^17^ In every cell, there are two major enzymes that play essential roles: Phosphatases and kinases. Phosphatases are the enzymes that can remove a phosphate group from protein, while kinases transfer a phosphate group to a target molecule. These two enzymes work together to regulate various signaling pathways in response to external stimuli, a vital function in the cell.^18^ The enzyme used in this study is tyrosine-protein kinase ABL1, a member of the kinase family. The ABL1 gene is involved in a number of biological processes including cell division, adhesion, differentiation, and stress response like DNA repair.^19,20^ The structure of the ABL1 protein can be divided into the SH3 domain, SH2 domain, and protein kinase domain.

Ligand-gated ion channels (LICs, LGICs) belong to the family of transmembrane ion-channel proteins that can open to enable ions such as Na^+^, K^+^, Ca^2+^, and Cl^–^ to pass through the membrane in response to the binding of a chemical messenger such as a neurotransmitter.^21^ This bidirectional functional cross-talk is crucial for vital cellular activities, including excitotoxicity in pathological and disease states like stroke and the fundamental dynamics of activity-dependent synaptic plasticity.^22^ Here, we chose the *N*-methyl-d-aspartate receptor (NMDAR) as a typical example of an ion-channel protein. NMDAR responds to the neurotransmitter glutamate, belonging to the glutamate receptor family, and is found in most excitatory synapses.^23^ Playing a critical role in synapse formation and plasticity, NMDAR is a prototypical allosteric machine with extensive extracellular N-terminal domains (NTDs). The NMDAR can offer allosteric regulation of important receptor characteristics that influence cognition and behavior.^24^

To perform the study, we first analyzed the binding free energy and root-mean-square deviation (RMSD) to see whether MD simulations can simulate the stable state in a dynamic process of our collected structures. Subsequently, we used the representative frame of the MD trajectory and small molecule–residue interaction energy profile to confirm our conclusion in the previous work. Moreover, we explored a binding pattern or preference between receptor–orthosteric ligand–PAM and receptor–orthosteric ligand–NAM. Finally, we analyzed the conformational changes of the whole structure after AM binding.

## 2 Results and Discussion

The results will be divided into four categories in this section: (1) MD simulation’s ability to sample and simulate the stable dynamic process of the collected structures; (2) orthosteric binding pocket comparisons between receptor–orthosteric ligand complex and receptor–orthosteric ligand–allosteric modulator complex; (3) allosteric modulator binding pattern; and (4) complex conformational changes in different states within the same receptor system.

### 2.1 Binding-Free Energies of All Complexes

Table S1 shows details of each complex with its ligand–protein binding free energy. As the principle of binding free energy calculation, a negative value means that the process of ligand–protein binding from their respective separate state to a combined complex state can occur spontaneously, and it means that the final complex status should be stable. A lower negative value indicates a strong binding affinity. From Table S1, we can see that all the binding free energies are negative. Such results, combined with the low RMSDs discussed in the next sub-section, mean that all the complexes in this study were under stable status during the MD simulations, which can serve as the basis of the analysis of the subsequent results. In addition to the total ligand–protein binding free energies, we also calculated the orthosteric/allosteric ligand–residue interaction free energy profiles (which will be discussed in later sub-sections) to identify the precise contribution of individual residues to the orthosteric binding and allosteric binding, and hence the corresponding hotspot residues.

### 2.2 RMSD Graphs of All Complexes

In addition to binding free energy, the RMSD values for the whole MD simulation process are also a common factor to evaluate the simulation quality. We regard a value under 5 Å for RMSD as a good score, representing the stable dynamic status. All the RMSD graphs are shown in Figures S1–S3 in the supplementary materials.

From Figure S1, it can be seen that most of the RMSD values were under 3 Å, indicating an ideal stable dynamic status of the β2 adrenoceptor system. No obvious fluctuation was observed, thus, the simulation of the β2 adrenoceptor system was successful (for the RMSD graph of β2 adrenoceptor-carazolol-NAM, the results were shown in our previous work.^10^ We used the trajectory files from it, so the RMSD data were consistent. But we performed a totally different post-MD analysis to generate new data in the next sessions in this study.).

In the RMSD graphs of the ABL1 system (Figure S2), results were even better than those in the β2 adrenoceptor. There was no apparent fluctuation since all the RMSD values reached a stable condition before 10 ns. From Figure S2, it can be observed that the RMSD value of ABL1 was always higher than that of the orthosteric ligand or the allosteric modulator, which might be related to the higher fluctuation caused by higher flexibility of an enzyme (protein) compared to the peptide or small molecule.

For the NMDAR system, all complexes shared the same agonist (glutamate), so only 3 RMSD graphs were generated for this study. Almost all the RMSDs of every content in this system are lower than 3 Å. Glutamate underwent fluctuation in Figure S3c, which might be because of the small molecular size from the amino acids. For a relatively large binding pocket, glutamate might have more flexibility during the simulation process. Although they were fluctuating in the second half of the simulation, the RMSD values of glutamate stayed around 3 Å, which can still be interpreted as a stable status of the complex.

As a summary of the binding free energy table and RMSDs graphs, we believed that the MD simulations performed in this study has the eligibility to simulate the stable dynamic process of the collected structures, which provides the foundation for all our analyses later.

### 2.3 Comparison of the Orthosteric Binding Pocket

**Figure 1:**
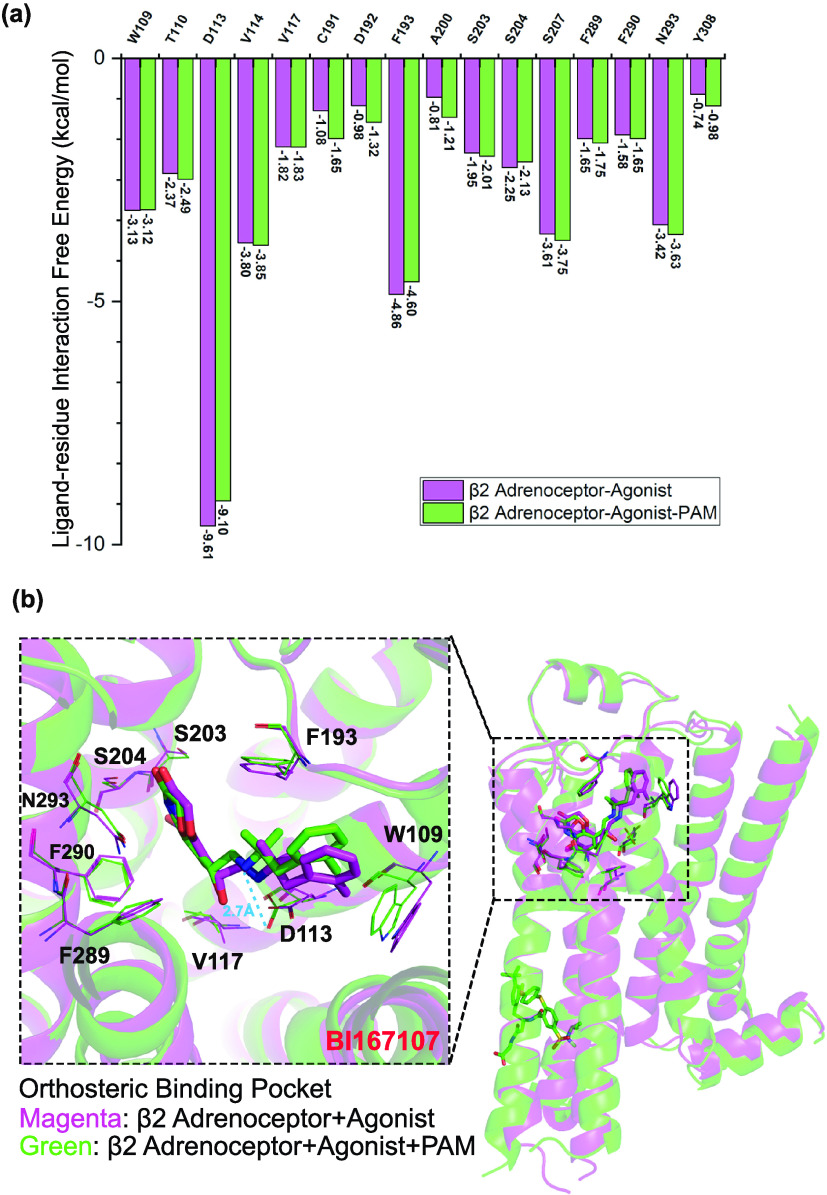
Orthosteric binding pocket comparison in β2 adrenoceptor–agonist–PAM after MD simulations. The color magenta represents the complex of β2 adrenoceptor and agonist BI167107 (PDB ID: *4LDE*); the color green represents the complex of β2 adrenoceptor–agonist BI167107-PAM Cmpd-6FA (PDB ID: *6N48*). The dashed light blue lines in the expanded view indicate a hydrogen bond between two linked atoms, and the blue number with Å shows the hydrogen bond length. (a) Ligand–residue interaction free energies between the residues of the β2 adrenoceptor and the agonist with/without PAM; (b) The aligned representative structures of β2 adrenoceptor plus agonist with/without PAM and their expanded view on the orthosteric binding pocket.

**Figure 2:**
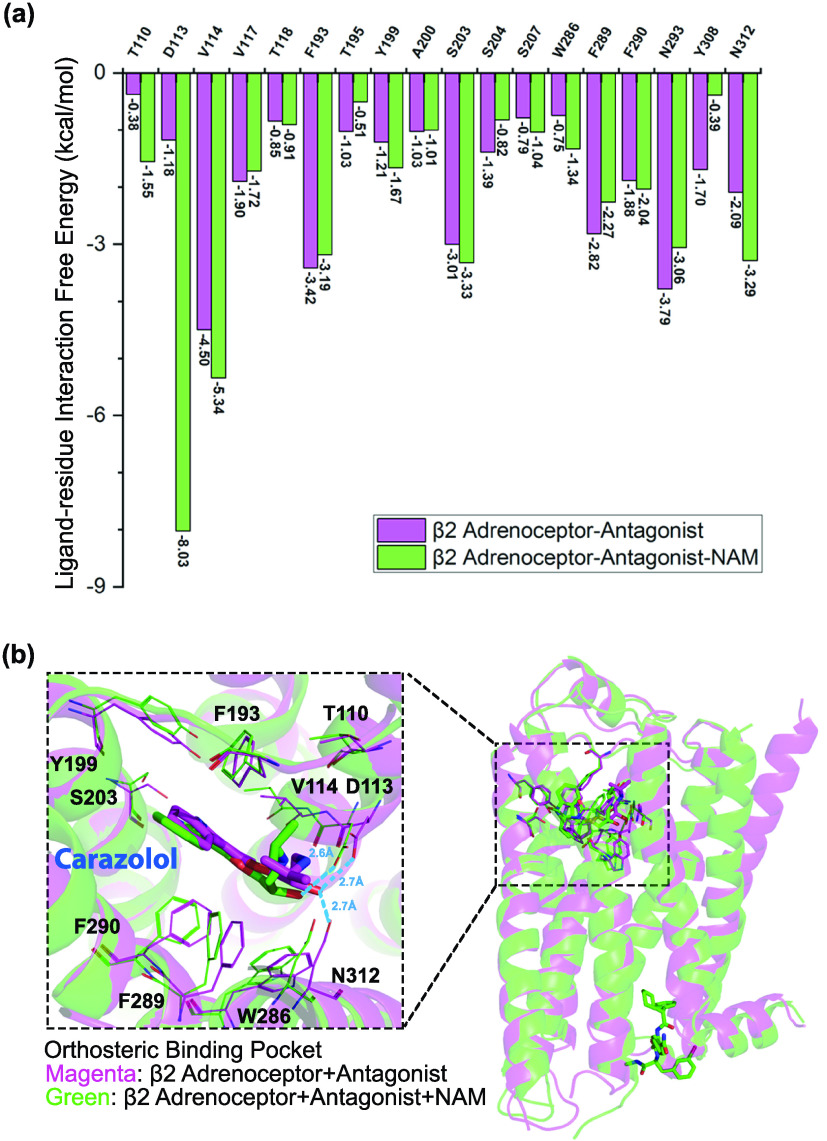
Orthosteric binding pocket comparison in β2 adrenoceptor–antagonist–NAM after MD simulations. The color magenta represents the complex of β2 adrenoceptor and antagonist carazolol (PDB ID: 2RH1); the color green represents the complex of β2 adrenoceptor–antagonist carazolol–NAM Cmpd-15PA (PDB ID: 5X7D). The dashed light blue lines in the expanded view indicate a hydrogen bond between two linked atoms, and the blue number with Å shows the hydrogen bond length. (a) Ligand–residue interaction free energies between the residues of the β2 adrenoceptor and the antagonist with/without NAM; (b) The aligned representative structures of β2 adrenoceptor plus antagonist with/without NAM and their expanded view on the orthosteric binding pocket.

**Figure 3:**
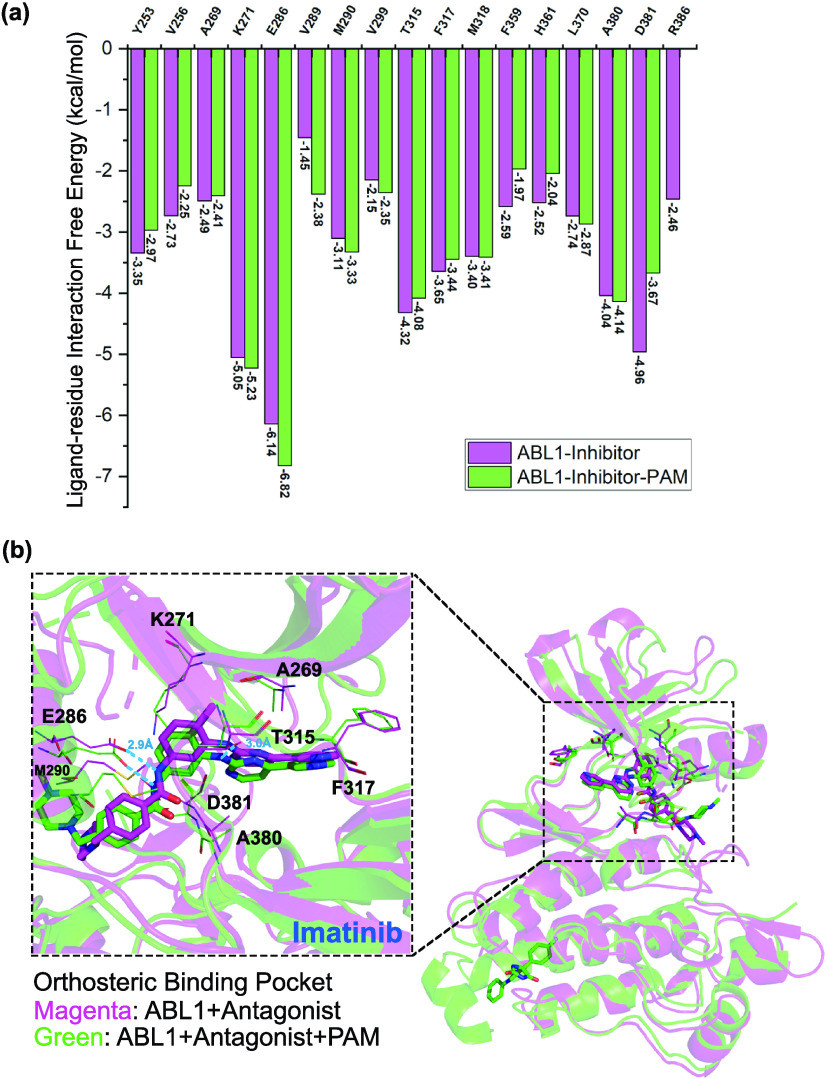
Orthosteric binding pocket comparison in ABL1–antagonist–PAM after MD simulations. The color magenta represents the complex of ABL1 and antagonist Imatinib (PDB ID: 2HYY); the color green represents the complex ABL1–antagonist Imatinib–PAM DPH (PDB ID: 3PYY). The dashed light blue lines in the expanded view indicate a hydrogen bond between two linked atoms, and the blue number with Å shows the hydrogen bond length. (a) Ligand–residue interaction free energies between the residues of ABL1 and the antagonist with/without PAM; (b) aligned representative structures of ABL1 plus antagonist with/without PAM and their expanded view on the orthosteric binding pocket.

**Figure 4:**
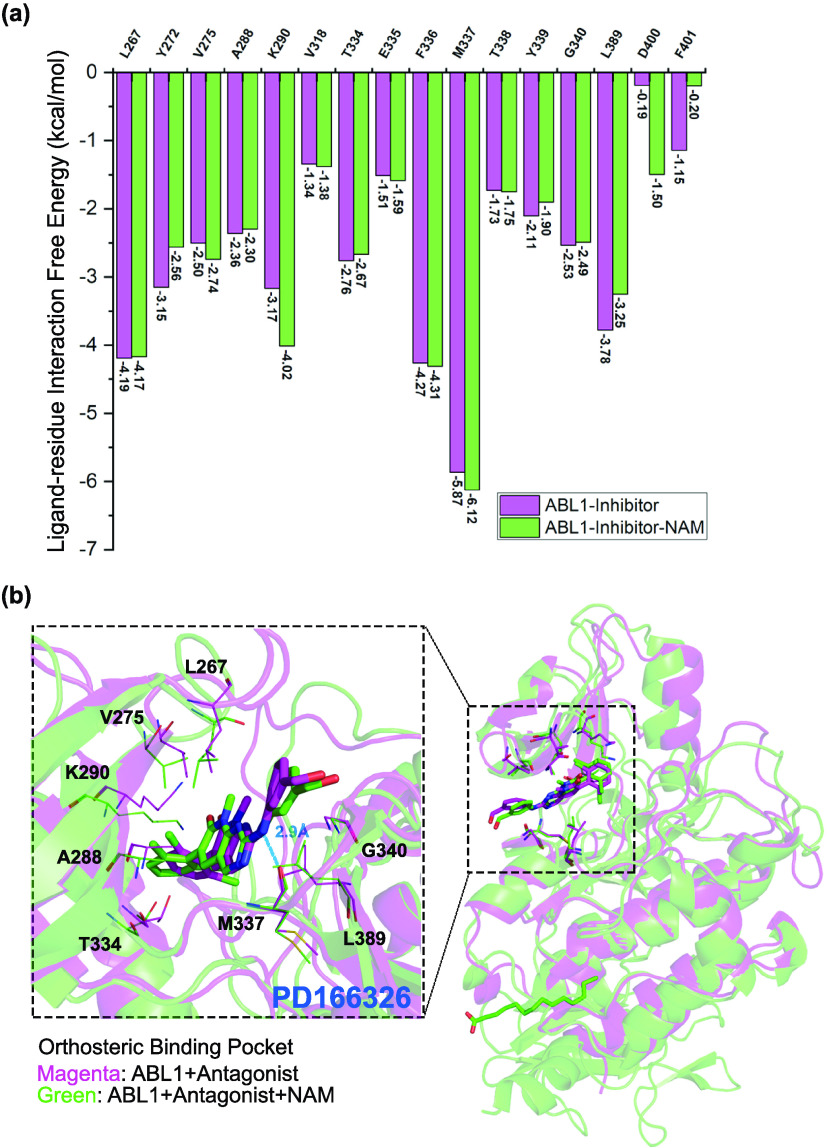
Orthosteric binding pocket comparison in ABL1–antagonist–NAM after MD simulations. The color magenta represents the complex of ABL1 and antagonist PD166326 (PDB ID: 2G2H); the color green represents the complex ABL1–antagonist PD166326–PAM myristic acid (PDB ID: 1OPK). The dashed light blue lines in the expanded view indicate a hydrogen bond between two linked atoms, and the blue number with Å shows the hydrogen bond length. (a) Ligand–residue interaction free energies between the residues of ABL1 and the antagonist with/without NAM; (b) The aligned representative structures of ABL1 plus antagonist with/without NAM and their expanded view on the orthosteric binding pocket.

**Figure 5:**
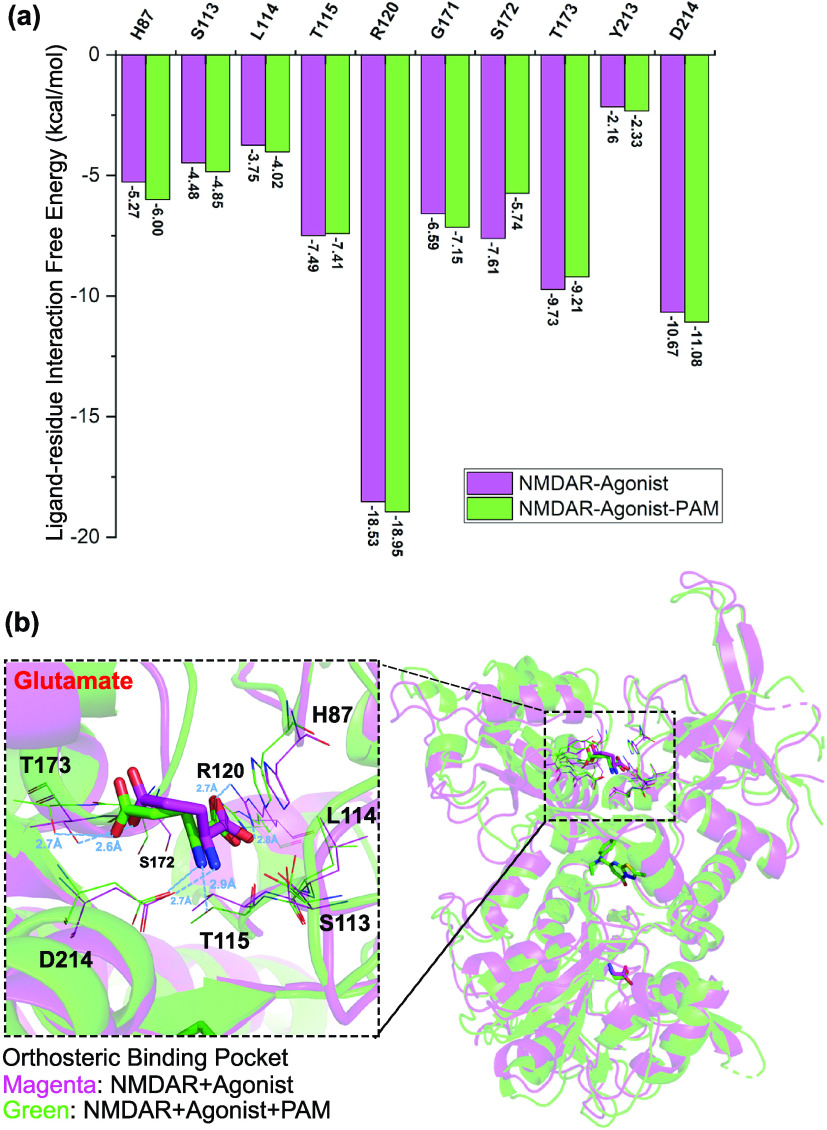
Orthosteric binding pocket comparison in NMDAR–agonist–PAM after MD simulations. The color magenta represents the complex of NMDAR and agonist glutamate (PDB ID: 5H8F); the color green represents the complex NMDAR–agonist glutamate-PAM GNE3419(PDB ID: 5H8H). The dashed light blue lines in the expanded view indicate a hydrogen bond between two linked atoms, and the blue number with Å shows the hydrogen bond length. (a) Ligand–residue interaction free energies between the residues of NMDAR and the agonist with/without PAM; (b) The aligned representative structures of NMDAR plus agonist with/without PAM and their expanded view on the orthosteric binding pocket.

**Figure 6:**
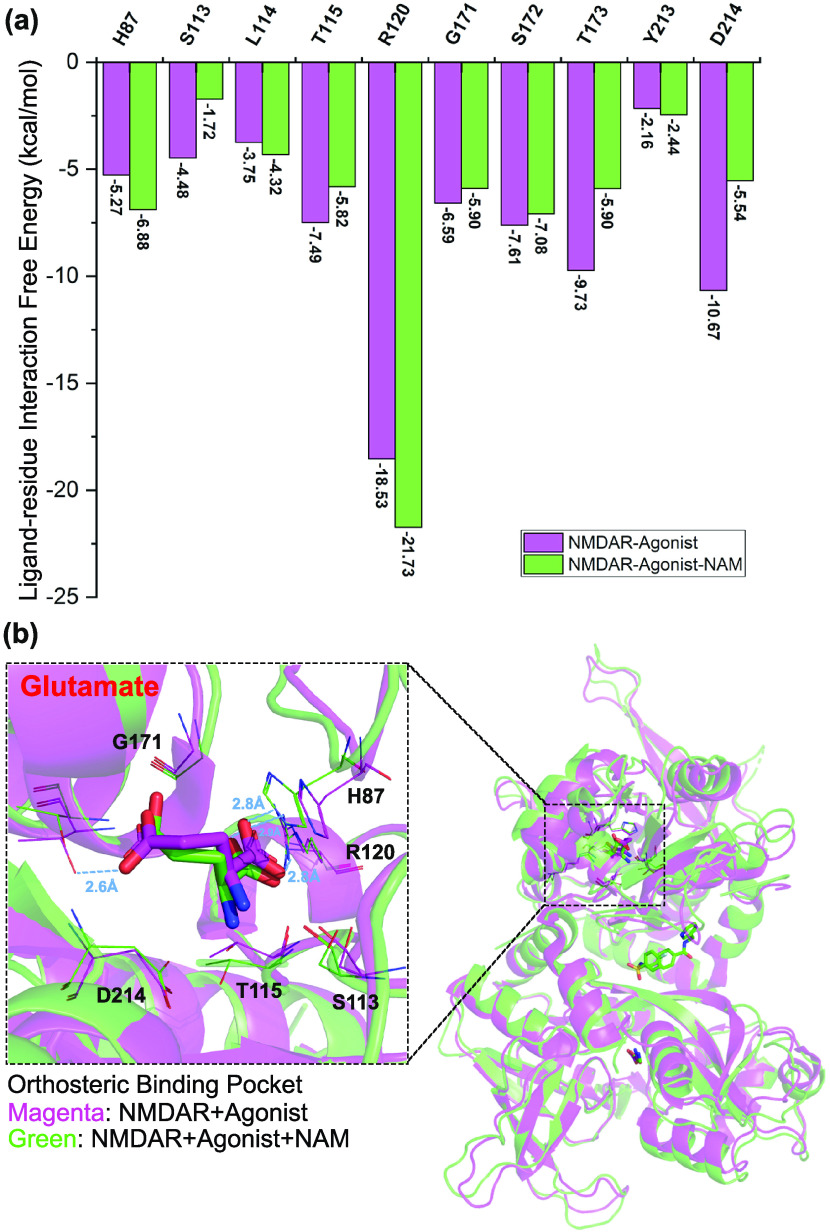
Orthosteric binding pocket comparison in NMDAR–agonist–NAM after MD simulations. The color magenta represents the complex of NMDAR and agonist glutamate (PDB ID: 5H8F); the color green represents the complex NMDAR–agonist glutamate–NAM Compound 6 (PDB ID: 5H8N). The dashed light blue lines in the expanded view indicate a hydrogen bond between two linked atoms, and the blue number with Å shows the hydrogen bond length. (a) Ligand–residue interaction free energies between the residues of NMDAR and the agonist with/without NAM; (b) The aligned representative structures of NMDAR plus agonist with/without NAM and their expanded view on the orthosteric binding pocket.

**Figure 7:**
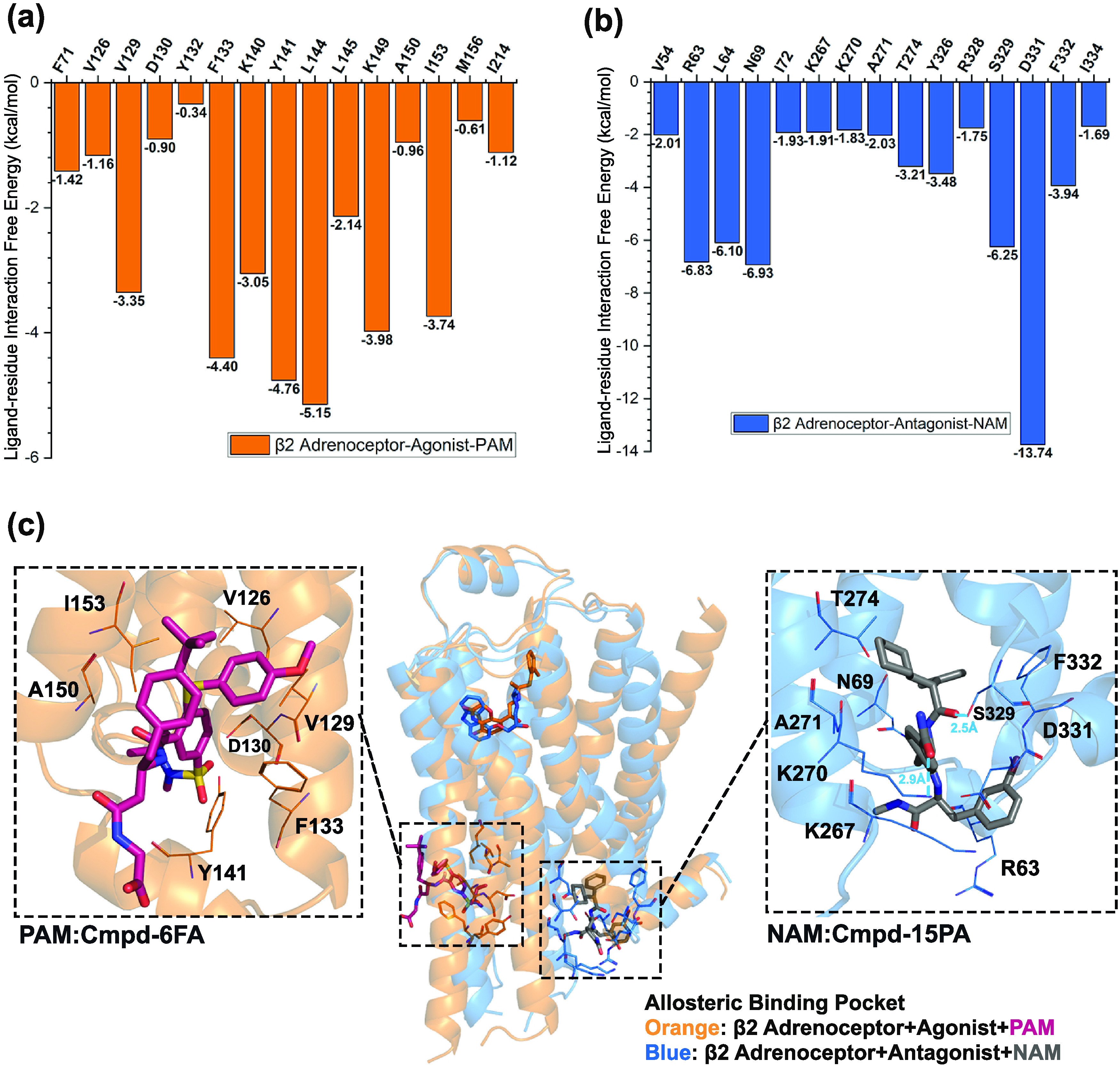
Allosteric binding pocket comparison in the β2 adrenoceptor system after MD simulations. The color orange represents the complex of β2 adrenoceptor–agonist BI167107 (PDB ID: 6N48); the color blue represents the complex of β2 adrenoceptor–antagonist (PDB ID: 2RH1); the color warm pink represents the PAM Cmpd-6FA; the color gray represents the NAM Cmpd-15PA. The dashed light blue lines in the expanded view indicate a hydrogen bond between two linked atoms, and the blue number with Å shows the hydrogen bond length. (a) The hotspot residues with the PAM–residue interaction free energies lower than −0.1 kcal/mol in the system of β2 adrenoceptor–Agonist–PAM; (b) The hotspot residues with the NAM–residue interaction free energies lower than −0.1 kcal/mol in the system of β2 adrenoceptor–antagonist–NAM; (c) The aligned representative structures of β2 adrenoceptor–agonist/antagonist–PAM/NAM and their expanded view on the allosteric binding pocket.

**Figure 8:**
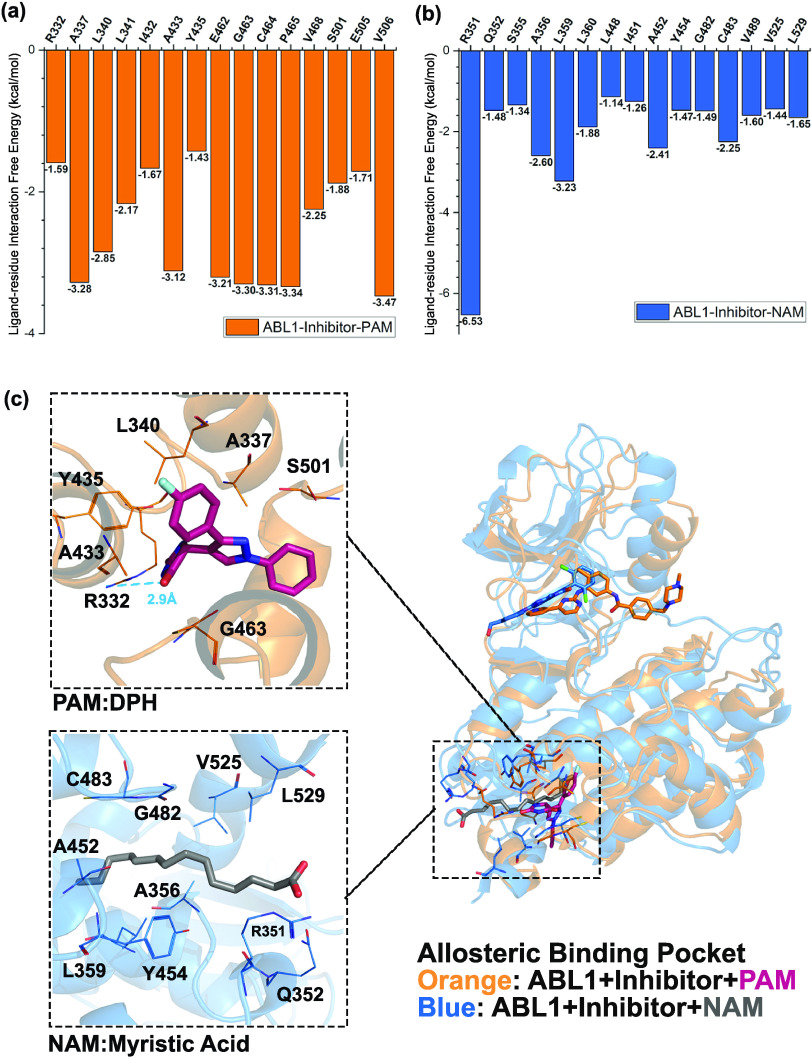
Allosteric binding pocket comparison in the ABL1 system after MD simulations. The color orange represents the complex ABL1–inhibitor imatinib (PDB ID: 3PYY); the color blue represents the complex ABL1–inhibitor PD166326 (PDB ID: 1OPK); the color warm pink represents the PAM DPH; the color gray represents the NAM myristic acid. Blue dashed lines in the expanded view indicate a hydrogen bond between two linked atoms and the blue number with Å shows the hydrogen bond length. (a) The hotspot residues with the PAM–residue interaction free energies lower than −0.1 kcal/mol in the system of ABL1–Inhibitor–PAM; (b) The hotspot residues with the NAM–residue interaction free energies lower than −0.1 kcal/mol in the system ABL1–Inhibitor–NAM; (c) The aligned representative structures of ABL1–inhibitor–PAM/NAM and their expanded view on the allosteric binding pocket.

**Figure 9:**
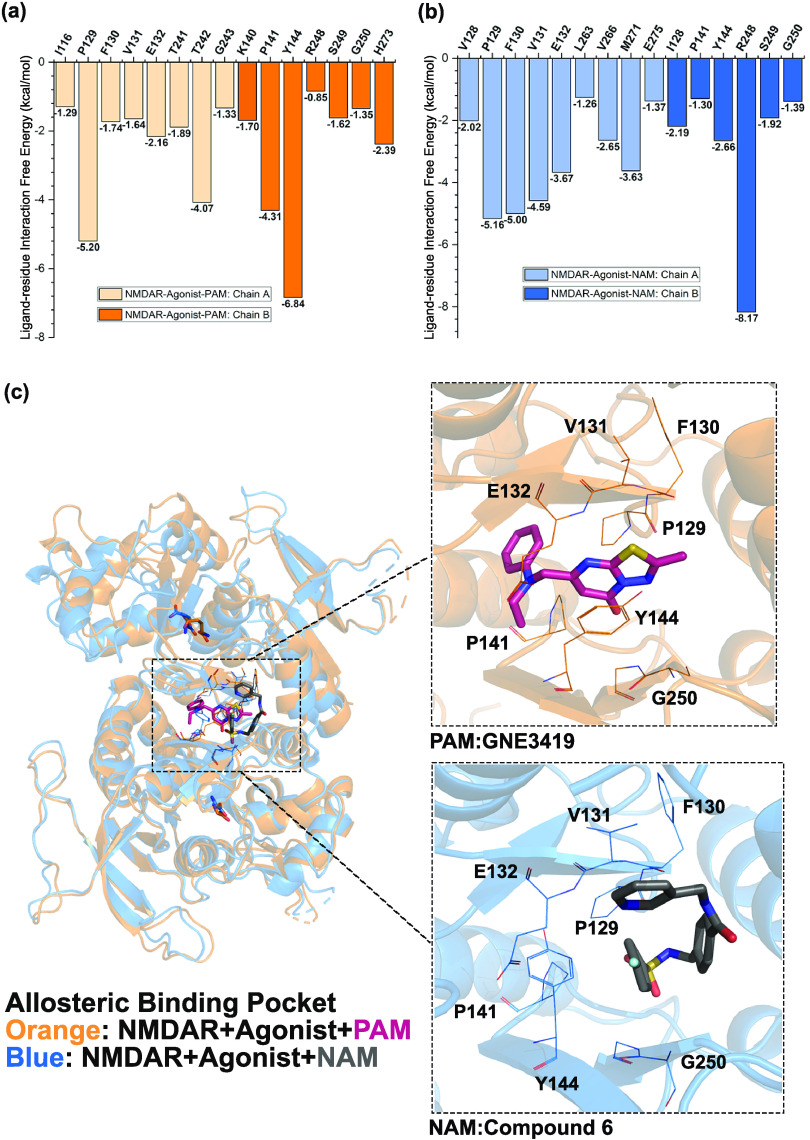
Allosteric binding pocket comparison in the NMDAR system after MD simulations. The color orange represents the complex NMDAR–agonist(-PAM) (PDB ID: 5H8H); the color blue represents the complex NMDAR–agonist(-NAM) (PDB ID: 5H8N); the color warm pink represents PAM GNE3419; and the color gray represents the NAM Compound 6. (a) The hotspot residues with the PAM–residue interaction free energies lower than −0.1 kcal/mol in the NMDAR–Agonist–PAM system; (b) The hotspot residues with the NAM–residue interaction free energies lower than −0.1 kcal/mol in the system of NMDAR–agonist–NAM; (c) the aligned representative structures of NMDAR–agonist–PAM/NAM and their expanded view in the allosteric binding pocket.

Based on the MCCS toolset and crystal structures, as described in the Introduction section, we concluded that the binding of the allosteric modulator will have an insignificant impact on the orthosteric binding pocket. In this part, comparisons of orthosteric binding pocket changes before/after the binding of the allosteric modulator will be performed using the representative frame of the complex structure obtained from the MD simulation. From the results of binding free energy and the RMSD value (all the complexes being studied through MD simulations in this study reached the equilibrium state in the simulating process), we calculated the average structure first, then we picked out the frame with the lowest RMSD difference compared with the average one as the representative frame to reflect the complex in a stable dynamic status. The ligand–residue interaction free energy decomposition results have also been added to quantify the binding status between the receptor residues and the orthosteric ligand. Here, we regarded the ligand–residue interaction energy lower than −0.1 kcal/mol as a strong interaction between the calculated residues and the small molecule/peptide ligand. In each panel (a) of Figures 1–6, residues with the ligand–residue interaction free energy below or around −1.0 kcal/mol in two complexes are added. Then we aligned them into one bar graph to create an evident contrast. For this purpose, we plotted some residues’ ligand–residue interaction energy values, which initially showed only once in the aligned bar graph. Then for the (a) and (b) panels of Figures 7–9, fifteen residues with the lowest ligand–residue interaction value are shown in the bar graph. Blue lines of dashes in the expanded view indicate a hydrogen bond between two linked atoms, and the blue number with Å shows the hydrogen bond length.

### 2.4 Comparison of the Orthosteric Binding Pocket in the β2 Adrenoceptor System

For the β2 adrenoceptor, we divided the structures into two groups: 1. Receptor–orthosteric ligand with/without PAM (referred to as PAM group); 2. Receptor–orthosteric ligand with/without NAM (referred to as the NAM group). For the PAM group, the orthosteric ligand is BI167107, an agonist with ultrahigh affinity reported by Ring et al.^25^ It can be seen in Figure 1a that the ligand–residue interaction value did not change much, indicating that all of the listed residues had a strong and similar contribution to the orthosteric binding pocket before/after PAM binding. As the residue, which had the strongest interaction with the orthosteric ligand in both complexes, Asp113^3.32^ formed a hydrogen bond with the nitrogen atom of the agonist. This is consistent with assay results from Strader et al. that Asp113^3.32^ plays a vital role in the binding affinity of β2 adrenoceptor.^26^ For the NAM group, the orthosteric ligand is carazolol, a partial inverse agonist binding with picomolar affinity to β2 adrenoceptor-T4L, which can reduce the basal activity of the receptor.^27^Figure 2a shows that most of the ligand–residue interaction values did not change much, except for Thr110, Asp113^3.32^, and Tyr308^7.34^. From Figure 2b, we can see the Thr110^3.29^ of both complexes nearly overlapped, Asp113^3.32^ of both complexes move parallelly for a small distance. However, the movement of Asp113^3.32^ and the pose change of carazolol make the residue–ligand distance smaller with the influence of NAM, thus causing a significant change in the residue–ligand interaction free energy (from −1.18 to −8.03 kcal/mol). Tyr308^7.34^ was reported by Woo et al.^28^ to be the key residue on β2 adrenoceptor, participating in specific interactions with the ligand to stabilize receptor conformations that enhance the receptor–Gs protein coupling, resulting in Gs-biased agonism. It is also worth mentioning that Asp113^3.32^, Val114^3.33^, and Asn312^7.38^ form hydrogen bonds with carazolol, as shown in the expanded view of the orthosteric binding pocket.

### 2.5 Comparison of the Orthosteric Binding Pocket in the ABL1 System

In the ABL1 system, we collected two pairs of crystal structures of the ABL1 receptor bound with the orthosteric antagonist, including the receptor–Imatinib with/without PAM and the complex receptor–PD166326 with/without NAM. As shown in Figure 3a, every pair of ligand–interacting residues had nearly the same ligand–residue interaction free energy, and most of these residues present fully overlapped conformation and the same orientation in Figure 3b, primarily indicating a minor change of the orthosteric binding pocket after PAM binds to ABL1. Among them, Glu286 was positioned to form a hydrogen bond with Imatinib in both complexes. PAM also did not alter the hydrogen bond between Thr315 and Imatinib. Thr315 had been reported to be the critical residue related to the bioactivity of orthosteric ligands of the ABL1 receptor by controlling the opening and closure of the P-loop and hinge in ABL1. Its T315I mutation not only broke the hydrogen bond but also introduced severe steric clashes between the orthosteric ligands and ABL1.^29^ Due to a slightly missing crystal structure of ABL1–inhibitor–PAM, Arg386 did not appear in this structure. Therefore, it did not have the calculated ligand–residue interaction free energy in Figure 3a. Except that, only Val289 and Asp381 had small changes in the interaction between the orthosteric ligand and ABL1. In the NAM group, scenarios were similar to the first group; the ligand–interacting residues in the orthosteric binding sites of the complexes of ABL1–inhibitor with/without NAM had similar ligand–residue interaction free energy, except for two pairs of residues, Asp400 and Phe401 (Figure 4a). It can be illustrated that after NAM is bound to the ABL1 receptor, the orthosteric ligand PD166326 is slightly reoriented to form a new interaction with Asp400 and move away from the previous interacting residue Phe401. Met337 contributed to a strong hydrogen bond with the orthosteric ligand. Lys290 also had a tiny change in the binding between the orthosteric ligand and ABL1.

To summarize, although the two orthosteric ligands were different and had different interactions with the ABL1 receptor, the binding of both NAM and PAM had a minor impact on the size and ligand–interacting residues of the orthosteric binding site.

### 2.6 Comparison of the Orthosteric Binding Pocket in the NMDAR System

For the NMDAR system, glutamate acts as the same agonist for both NMDAR–agonist–PAM and NMDAR–agonist–NAM complex. Endogenous glutamate from cells was present at a concentration that caused submaximal activation, allowing substances that increase NMDAR Ca^2+^ influx to be identified.^30^ Although NMDAR required the presence of glycine to assist the glutamate for effective channel activation,^31^ we regard glutamate as the major agonist to calculate the ligand–residue interaction free energies since only one ligand may be selected for calculation after the simulation, and the location of the independent glycine is away from the major orthosteric binding pocket. For the PAM group, it can be seen in Figure 5a that the ligand–residue interaction free energy of each ligand–interacting residue is barely different between with/without PAM results. In Figure 5b, glutamate and residues within the orthosteric binding pocket nearly overlapped, indicating an insignificant conformational change of the orthosteric binding pocket after PAM binds to NMDAR. Thr115, Arg120, Thr173, and Asp214 formed hydrogen bonds between glutamate and NMDAR, which is also consistent with the search results of Sadaf et al.^32^ For the NAM group, the situation is slightly different in that after the simulation, the position of glutamate has changed with a mild shift (Figure 6b), leading to the ligand–residue interaction free energy difference between with/without NAM structures. As glutamate is a small molecule, its flexibility in a comparatively big binding pocket is acceptable. While the value gap in the NAM group is more obvious than that in the PAM group, the top-ranking ligand–interacting residues shown in Figure 6a are completely the same as shown in Figure 5a. All of the ligand–residue interaction free energy was lower than −1.0 kcal/mol, which represented strong interaction between the glutamate and NMDAR. His87, Arg120, and Gly171 still form hydrogen bonds between glutamate and NMDAR.

Overall, the orthosteric binding pattern such as key binding residues or binding pose will not be obviously influenced by the PAM/NAM binding in the NMDAR system. The results of 3 different receptor–ligand–allosteric modulator systems are consistent with our previous research that the binding of the allosteric modulator will have an insignificant impact on the orthosteric binding pocket.^10^

### 2.7 Binding Pattern of the Allosteric Binding Pocket

During our previous research on the allosteric modulator design or allosteric modulator binding position search, we found that people tend to have the intuition that the pocket of these two modulators (PAM and NAM) will not be located in the same position due to their opposite effects. Here, we figured out whether there is a binding pattern of the allosteric binding pocket and found more details of the allosteric binding pocket in each of the chosen systems.

From the residues in the bar graph and the aligned structures of the two complexes (Figure 7), it was obvious that these two allosteric binding pockets are not in the same location. The PAM is located in the TMs 2, 3, 4, and intracellular loop (ICL) 2 (ICL2), while the NAM is located within the TMs 1, 2, 6, 7, ICL1, and helix 8. The PAM Cmpd-6FA was reported by Liu et al.^33^ This allosteric modulator has strong positive cooperativity with orthosteric agonists and transducers (β-arrestin and G proteins). It potentiates agonist-induced adenosine-3′,5′-cyclic monophosphate synthesis as well as β-arrestin recruitment to the β2 adrenoceptor.^34^ Cmpd-6FA consists of four chemical groups: the core scaffolds, *N*-methylpropan-2-amine (R1), 4-methoxybenzene (R2), and tert-butylbenzene (R3). The R1, R2, and R3 chemical moieties engage with the cytoplasmic, membrane-proximal, and membrane-embedded interfaces of the β2 adrenoceptor, respectively. The core scaffold connected the membrane-proximal and cytoplasmic surfaces and served as a hub for the R1, R2, and R3 groups to stretch into their respective binding surface characteristics. For detailed interactions, Cmpd-6FA formed a strong interaction with Phe133^3.52^ and Tyr141^ICL2^, which showed consistent results with Liu’s work.^33^ From their work, the side chains of the β2 adrenoceptor coupled to Cmpd-6FA, and the two residues Phe133^3.52^ and Tyr141^ICL2^ were rotated to offer excellent form complementarity with the allosteric ligand. The Cmpd-6FA’s acetate group was chemically added to improve compound solubility, making limited contact with the β2 adrenoceptor and pointing toward the solvent in the crystal structure.

We proceeded by looking at the NAM binding pocket. Cmpd-15 crossed the plasma membrane and bound to the β2 adrenoceptor’s intracellular surface, according to biochemical and pharmacological analysis. To understand how Cmpd-15 stabilizes the inactive state and inhibits coupling to G proteins and β-arrestins, a crystal structure of the β2 adrenoceptor bound to Cmpd-15 is reported by Liu et al.^33^ In their work, a molecule of carboxylic acid functionalized poly(ethylene glycol) was added to the location used for linking the molecule to its DNA tag to increase Cmpd-15 occupancy in the crystalline β2 adrenoceptor. The modified Cmpd-15 poly(ethylene glycol)-carboxylic acid derivative (referred tovas Cmpd-15PA) has a similar orthosteric agonist binding affinity compared to the original Cmpd-15.

Our results from the MD simulations were consistent with Liu’s work that Cmpd-15PA forms strong interactions with Ser329^8.47^, Asp331^8.49^, and Asn69^2.40^, and with the backbone carbonyl of Arg63^ICL1^. Cmpd-15PA’s bromophenyl ring destroys a salt bridge between Arg63^ICL1^ in ICL1 and Asp331^8.49^ in Helix 8, allowing a new salt bridge to develop between Lys267^6.29^ at the end of TM6 and Asp331^8.49^ in Helix 8. Cmpd-15PA is trapped in its binding pocket by this newly created salt bridge and forms hydrogen bonds with Lys270^6.32^ and Ser329^8.47^. From the work of Rasmussen et al.^35^ on the structure of the β2 adrenoceptor–Gs protein complex, we suggested that Cmpd-15PA will sterically clash with the C-terminal α5 helix of the G-protein Gs, blocking the binding of the G-protein with the β2 adrenoceptor. Thus, Cmpd-15PA can further strengthen the antagonistic effect of an antagonist.

Although the selected 15 residues with the lowest ligand–residue interaction values did not overlap because of the selection standard (not all residues with MMGBSA value lower than −0.1 kcal/mol are shown in the figure), an obvious overlap of both the allosteric binding pockets and allosteric modulators between the ABL1–inhibitor–PAM complex and the ABL1–inhibitor–NAM complex can be observed in Figure 11c.

Then for the ABL1 system, the PAM used in this part is DPH(5-[3-(4-fluorophenyl)-1-phenyl-1H-pyrazol-4-yl]-2,4-imidazolidinedione or 5-(1,3-diaryl-1H-pyrazol-4-yl)hydantoin), which stimulates c-Abl activation through enzymatic and cellular action. DPH binds to the myristoyl binding site and hinders the formation of the bent conformation of the αI helix through steric hindrance. It is the first cell-permeable, small-molecule c-Abl activation chemical.^36^ In this study, DPH forms a hydrogen bond with Arg332.

The NAM used in this part is myristic acid, which can trigger the conformational change of ABL1 by binding with the protein to the base of the kinase domain. The myristoyl group is anchored to a hydrophobic pocket between PKA’s distinctive N-terminal helix and the kinase domain’s two lobes and can help in stabilizing the assembled and inactive conformation of c-Abl. The blue structure used in this part (Figure 8c), myristoylated c-Abl1–531 bound to PD166326 at 3.4 Å resolution, is reported by Nagar et al..^37^ Arg351 is then calculated as the most critical residue in the allosteric pocket in this study.

Lastly in the NMDAR system, the bars in Figure 9a,b have been divided into light orange/blue and dark orange/blue since there are two chains in the NMDAR. Each chain has its independent order sequence. Looking at the allosteric binding pocket, not only do the two different allosteric modulators partially overlap in the allosteric binding pocket as observed in Figure 9c but also some of the selected residues shown in the ligand–residue interaction free energy bar graphs (Figure 9a,b) are the same, which indicates that the allosteric binding pockets between NMDAR–agonist–PAM and NMDAR–agonist–NAM complex can be regarded as the same binding pocket from both structure and calculation aspects.

The PAM, GNE3419, has the selectivity for GluN2A subunit-containing NMDARs and can increase the normal synaptic activation of NMDARs without leading to activation in the absence of stimulation.^30^ Pro129 and Thr242 in chain A as well as Pro141 and Tyr144 in chain B are additional essential residues for the interaction within the allosteric binding pocket based on the ligand–residue interaction free energy.

The NAM used in this study, compound 6, was first synthesized by Bettini et al.,^38^ and then was reported by Hackos et al.^30^ as a 2.5 Å X-ray structure. Pro129, Phe130, and Val131 in chain A and Arg248 in chain B have strong interactions between Compound 6 and NMDAR.

### 2.8 Conformational Changes of the Complex in Different States

**Figure 10:**
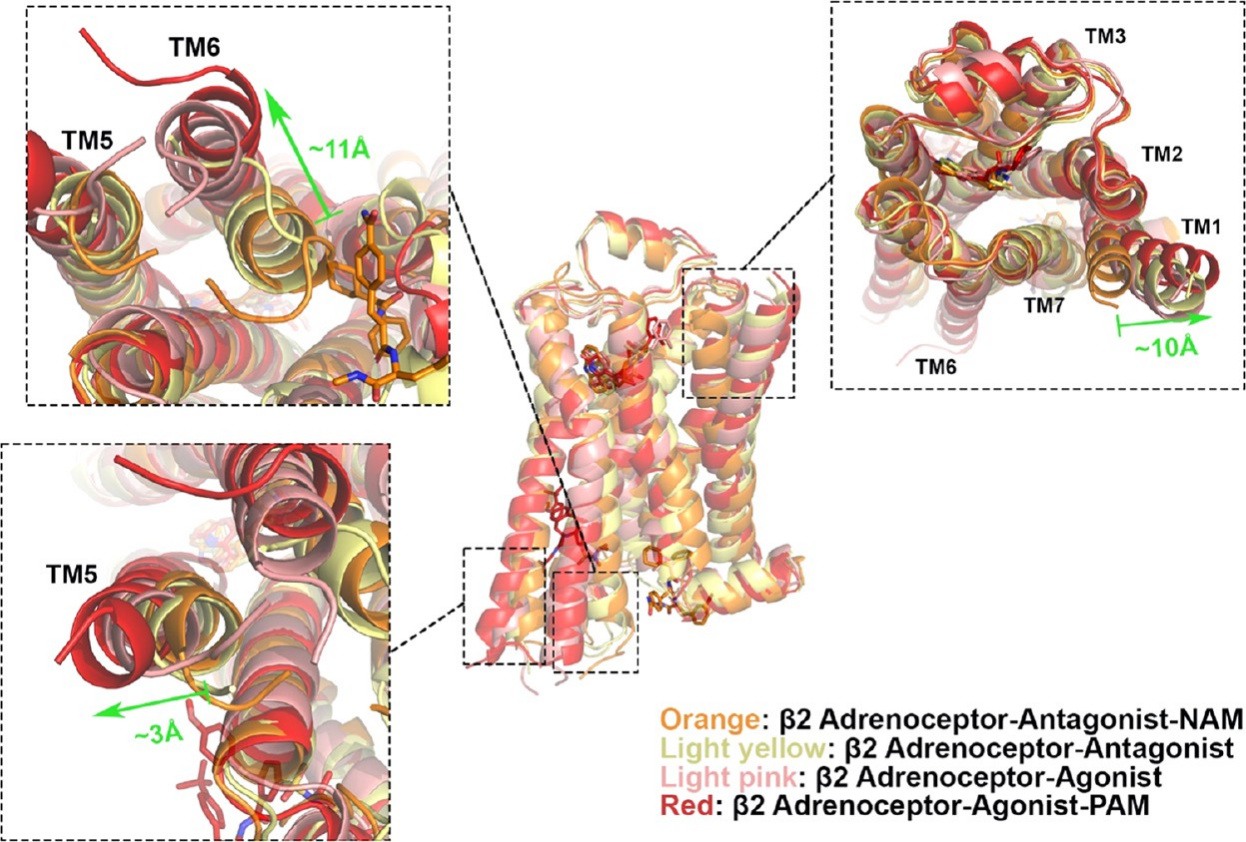
Conformational changes of β2 adrenoceptor complexes in different states compared by aligning the representative frames of MD simulations. The color orange represents the complex of β2 adrenoceptor–antagonist–NAM (initial MD conformation from PDB ID 5X7D); the color light yellow represents the complex β2 adrenoceptor–antagonist (initial MD conformation from PDB ID 2RH1); the color light pink represents the complex β2 adrenoceptor–agonist (initial MD conformation from PDB ID 4LDE); the color red represents the complex β2 adrenoceptor–agonist–PAM (initial MD conformation from PDB ID 6N48). The offset distances are marked with green arrows in the figure, and the length units are Å.

**Figure 11:**
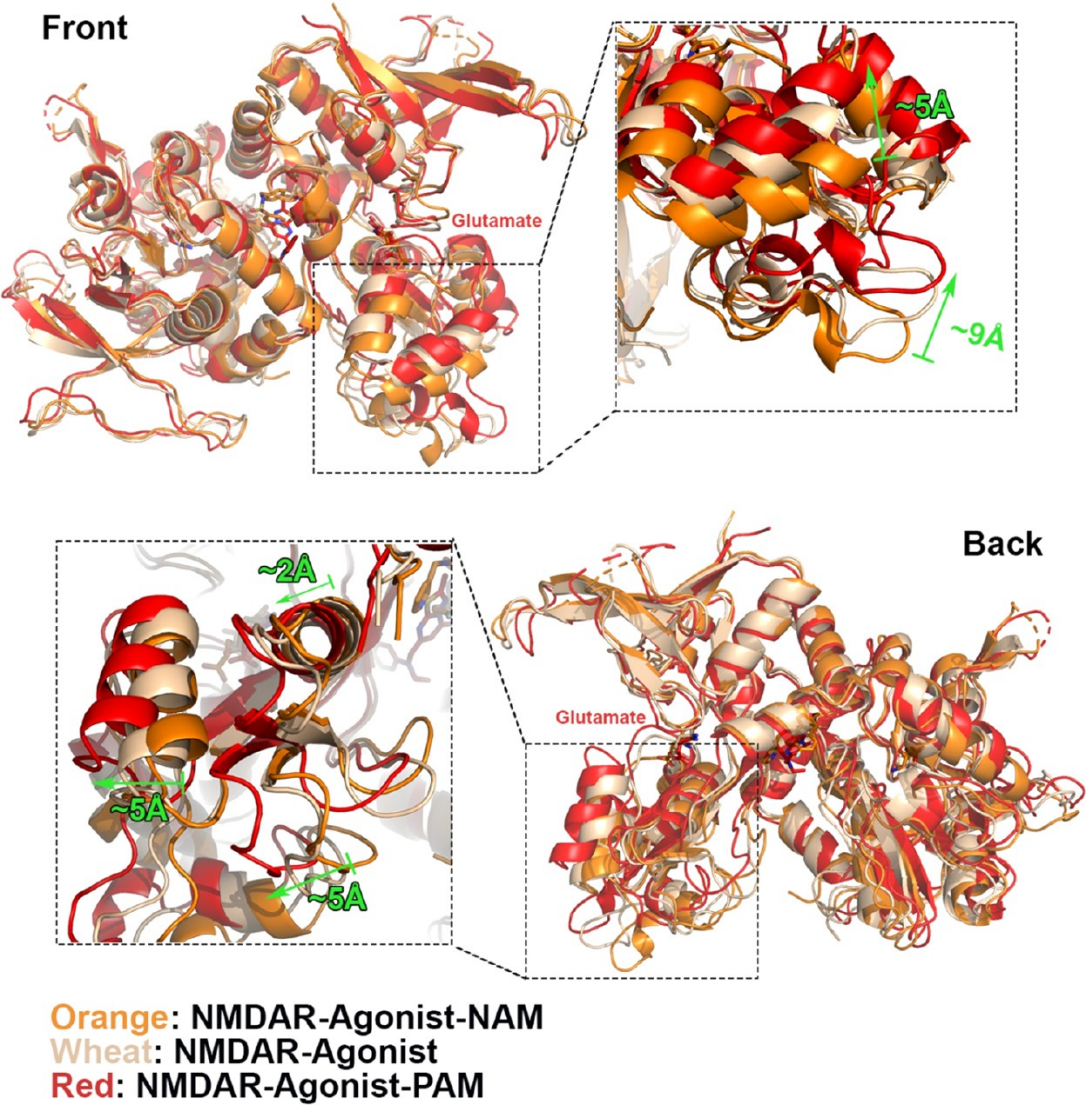
Conformational changes of NMDAR complexes in different states compared by aligning the representative frames of MD simulations. The color orange represents the complex NMDAR–agonist–NAM (initial MD conformation from PDB ID: 5H8N); the color wheat represents the complex NMDAR–agonist (initial MD conformation from PDB ID: 5H9F); the color red represents the complex NMDAR–agonist–PAM (initial MD conformation from PDB ID: 5H8H). The offset distances are marked with green arrows in the figure and the length units are Å.

Obvious and regular conformational changes from inactive states to active states were observed only in the β2 adrenoceptor and the NMDAR system, so here, only 2 systems of complexes are displayed for discussion (Figure 10, Figure 11).

To further visualize the difference between the inactive and active states of the β2 adrenoceptor, we aligned the four representative structures in one figure: β2 adrenoceptor–antagonist–NAM; β2 adrenoceptor–antagonist; β2 adrenoceptor–agonist–PAM; and β2 adrenoceptor–agonist. These four structures were collected; representative frames from different MD simulations for each system respectively and then were aligned in one figure for a more straightforward vision. Slight wobbles were common in MD simulations, here, in this part only regular conformational changes based on the characteristics of the four complexes are discussed as an insight for further study.

The distance marked in the figures is from β2 adrenoceptor–agonist–PAM to β2 adrenoceptor–antagonist–NAM, which can be regarded as the quantified conformational change between an enhanced active state and an enhanced inactive state. As depicted in the expanded view of the aligned structures, TM6 underwent a massive outward movement (about 11 Å). The inward conformations of 2 inactive states may be caused by the inclination to form a stable pocket with NAM Cmpd-15PA. Then with the movement of TM6, TM5 subsequently moved outwardly for a small distance (about 3 Å). From the top view of the aligned structures, it can be seen that for the upper part of TM1, there was about 10 Å of outward movement from an enhanced inactive state to an enhanced active state. However, two structures without an allosteric modulator did not experience a substantial conformational change. This is because its distance from the orthosteric ligands, TM1 of the β2 adrenoceptor is less important for the orthosteric binding pocket than TMs 2, 3, 5, 6, and 7. As a result, the conformational shift in TM1 will not have an inverse impact on the preceding finding.

The NMDAR complexes all shared the same agonist: glutamate, so we aligned three structures (receptor–agonist; receptor–agonist–PAM; receptor–agonist–NAM) to check the conformational changes in different states. Due to the orthosteric ligand-binding pocket belonging to the GluN2A ligand-binding domain (LBD),^39^ we focused on the conformational changes of this part from the front and back. As for the front view of the aligned structures in Figure 11, we can see that the amplified short helixes and the loops at the bottom moved upwardly when the complex state is from depressed activated to enhanced activated. The offset distances were about 5 and 9 Å, respectively. At the backside of the aligned structures, there were three significant movements. The amplified helixes and loops shift outwardly from the depressed activated state to the enhanced activated state, and the offset distance was about 2, 5, and 5 Å, respectively. In summary, the bottom section of NMDAR’s LBD, where the mentioned shift occurred intensively, undergoes an upward and outward shift during the gradually activating process, which might impact the sequent signaling transduction to the transmembrane domain (TMD) of NMDAR.

## 3 Conclusions

In our previous study, we worked on the crystal structures of the complex and used the MCCS toolset to quantify the interaction between the receptor and small molecule/peptide. To further confirm our previous conclusion drawn from static structures that binding of the allosteric modulator will have an insignificant impact on the orthosteric binding pocket (however, this does not mean the whole structure of the complex will remain the same), MD simulations were applied to the present work to predict a stable dynamic process of the receptor–orthosteric ligand–allosteric modulator complex and explore the conformational changes within the orthosteric/allosteric binding pocket. We prepared structures in three different receptor systems: β2 adrenoceptor system, tyrosine-protein method ABL1 system, and NMDAR (*N*-methyl-d-aspartate receptor) system. For the basis of this study, we ensured that MD simulation results used in this study can reflect a stable dynamic status of the originally collected structures. For this, the binding free energy table of complexes and RMSDs graphs were generated. Then, the representative frames of MD simulation results were collected to make a comparison between receptor–orthosteric ligand complexes and receptor–orthosteric ligand–allosteric modulator complexes. The comparison results showed that MD simulation results did not show a significant change with the results from the crystal structure for relatively big molecules or peptides. Due to the flexibility of small molecules like glutamate, the ligand–residue interaction free energy results of MD simulation on the receptor and small molecule may have been influenced.

Moreover, during the previous research on the allosteric modulator design and development, we observed through intuition that PAM and NAM should not locate at the same binding site because of their opposite effects. Comparing the allosteric binding pockets of three systems, we found that there should be no preference for the allosteric binding pocket position. Even if a PAM is determined to bind to a receptor at a specific site, one should not immediately disregard the chance that NAM can also bind at the same site. Finally, to investigate the conformational change, we aligned four different β2 adrenoceptor structures and three NMDAR structures in different states. For β2 adrenoceptor, the aligned results demonstrated that from an enhanced inactive state to an enhanced active state, TMs 1, 5, and 6 all moved outwardly gradually, among which TM 6 experienced the most significant conformational change (about 11 Å). For NMDAR, the bottom section of NMDAR’s LBD experienced an upward and outward shift during the gradually activating process. In summary, our study may provide insights into the receptor–orthosteric ligand–allosteric modulator study using MD simulations. This study may also help further in the design and development of allosteric modulator drugs.

## 4 Methods and Materials

### 4.1 MD Simulations

The receptors complexed with ligands were set up for MD simulations. All initial configurations were collected directly from the crystal structures published in the Protein Data bank^8^ (Table 1). Each system was put into a 0.15 M NaCl solution. For the systems of ABL1 and NMDAR, each complex was solvated in a cubic water box with the thickness of the water shell of at least 12 Å. The initial simulation boxes were 100 × 100 × 100 Å^3^ for ABL1 and 116 × 116 × 116 Å^3^ for NMDAR size. As to the GPCR protein β2 adrenoceptor, the bilayer membrane was constructed using the web application CHARMM-GUI (www.charmm-gui.org) with a size of around ∼128 × 110 × 128 Å^3^.^40−42^ The AMBER ff14SB force field^43^ was applied to proteins. The TIP3P (transferable intermolecular potential with three points) water model was used to handle water molecules.^44^ The semi-empirical with bond charge correction (AM1-BCC) approach was used to calculate the partial atomic charges of ligands.^45,46^ The other force field parameters were obtained from GAFF in AMBER16.^46^ The ANTECHAMBER module was used to create ligand residue topologies.^47^

The MD simulations used the PMEMD.mpi and PMEMD.cuda modules in the AMBER16^48−50^ package. The systems were first subjected to a series of reduction processes to minimize steric clashes. Then, during the heating step, each system was gradually heated from 0 to 300 K and maintained at 300 K during the succeeding equilibrium and production stages. For the heating, equilibrium, and complete production stages, a time step of 2 femtoseconds (fs) was used. A periodic boundary condition was used to keep the temperature and pressure constant in the ensembles. With a pressure relaxation time of 1 picosecond (ps), the pressure was set at 1 atm and adjusted by the anisotropic (*x*-, *y*-, *z*-) pressure scaling technique. Langevin dynamics with a collision frequency of 2 ps^–1^ were used to control the temperature.^51,52^ The particle mesh Ewald (PME) method^53,54^ was adopted to handle long-range electrostatics and a 10 Å cutoff was set to treat real-space interactions. All covalent bonds involving hydrogen atoms were constrained with the SHAKE algorithm.^55^ Each system was subjected to a 100 ns MD simulation, with the simulated systems’ trajectory being stored every 100 ps. For the representative frame of each MD simulation, we calculated the average structure from the saved trajectory first, then we picked out the frame with the lowest RMSD difference compared with the average one as the representative frame to reflect the complex in a stable dynamic status.

The calculation of the binding free energy for the binding of the ligand with a receptor to form a complex was based on the following equation
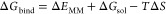
1where −*T*Δ*S* stands for the change in conformational entropy brought on by ligand-binding, Δ*G*_sol_ stands for the change in the solvation-free energy, and Δ*E*_MM_ is the change in molecular mechanics (MM) energy. Internal energies *E*_int_ (bond, angle, and dihedral energies), electrostatic energies E_ele_, and Van der Waals interaction energies *E*_VDW_ are some of the energy terms that make up *E*_MM_. *G*_sol_ can be further divided into two components (eq 2): the nonpolar contribution, which is characterized by the solvent-accessible surface area (SASA) (eq 3, where γ and *b* are a scaling and a constant parameter, respectively), and the polar contribution (electrostatic solvation energy), which can be calculated by either the Poisson–Boltzmann (PB) continuum solvation model or the Generalized Born (GB) surface area method:^56,57^
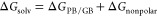
2
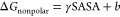
3

The entropy change −*T*Δ*S* was calculated by the weighted solvent-accessible surface area (WSAS) method^58^ in this study.

The free energy change of binding in eq 1 can be calculated between the ligand and the whole receptor (ligand–receptor binding free energy) and can be specifically calculated between the ligand and each of the residues in the receptor (ligand–residue interaction free energy). In this study, the binding free energy between a ligand and a whole receptor was calculated by the MMPBSA method using the Delphi program (http://compbio.clemson.edu/delphi),^59^ and the ligand–residue interaction free energy decomposition was calculated by the MMGBSA method implemented in AMBER Tools.

### 4.2 Research Systems

To investigate the molecular dynamics of the different receptor–orthosteric ligand–allosteric modulator complexes, we downloaded the structures from the Protein Data Bank (https://www.rcsb.org/). We then deleted the unnecessary molecules or sequences (for example, water molecules or G-protein). Only the receptor, orthosteric ligand, and allosteric modulator were used for the MD simulation. Then, we split the receptor–orthosteric ligand–allosteric modulator complex into independent files. For receptors with only the orthosteric ligand, we had two PDB. files to run the simulation, and for receptors with both an orthosteric ligand and an allosteric modulator, we had three PDB. files to run the simulation. During the simulation, the allosteric modulator and receptor were treated as a single entity to study the differences following orthosteric ligand binding.

## Supporting Information Available

Figure S1 shows the RMSDs of the β2 adrenoceptor system. Figure S2 shows the RMSDs of the ABL system. Figure S3 shows the RMSDs of the NMDAR system. Figure S4 shows the aligned structures of 4 ABL1 complexes. Table S1 shows the binding free energy of all complexes (PDF)
