# Supplementary material for: Molecular Modeling Study of a Receptor–Orthosteric Ligand–Allosteric Modulator Signaling Complex
Source: ACS Chem Neurosci. 2023 Jan 24;14(3):418–34. doi: 10.1021/acschemneuro.2c00554 (PMC10032570; doi:10.1021/acschemneuro.2c00554)
Supplement: Supplementary file 1 — cn2c00554_si_001.pdf [file cn2c00554_si_001.pdf]

Supporting Information

Molecular Modeling Study of Receptor-Orthosteric Ligand-Allosteric Modulator Signaling Complex

Chen Jiang[1], Xibing He[1], Yuanqiang Wang[2], Chih-Jung Chen[1], Yasmin Othman[1], Yixuan Hao[1], Jiayi Yuan[1], Xiang-Qun Xie[1]*, Zhiwei Feng[1]*

[1]Department of Pharmaceutical Sciences and Computational Chemical Genomics Screening Center, Pharmacometrics & System Pharmacology (PSP) PharmacoAnalytics, School of Pharmacy; National Center of Excellence for Computational Drug Abuse Research; Drug Discovery Institute; Departments of Computational Biology and Structural Biology, School of Medicine, University of Pittsburgh, Pittsburgh, Pennsylvania 15261, United States

[2]School of Pharmacy and Bioengineering, Chongqing University of Technology, Chongqing, 400054, China; Chongqing Key Laboratory of Medicinal Chemistry and Molecular Pharmacology, Chongqing, 400054, China; Chongqing Key Laboratory of Target Based Drug Screening and Effect Evaluation, Chongqing, 400054, China.

* To whom correspondence should be addressed. Tel: 412-383-5276; Fax: 412-383-7436; Email: xix15@pitt.edu. Tel: 412-419-4896; Email: zhf11@pitt.edu

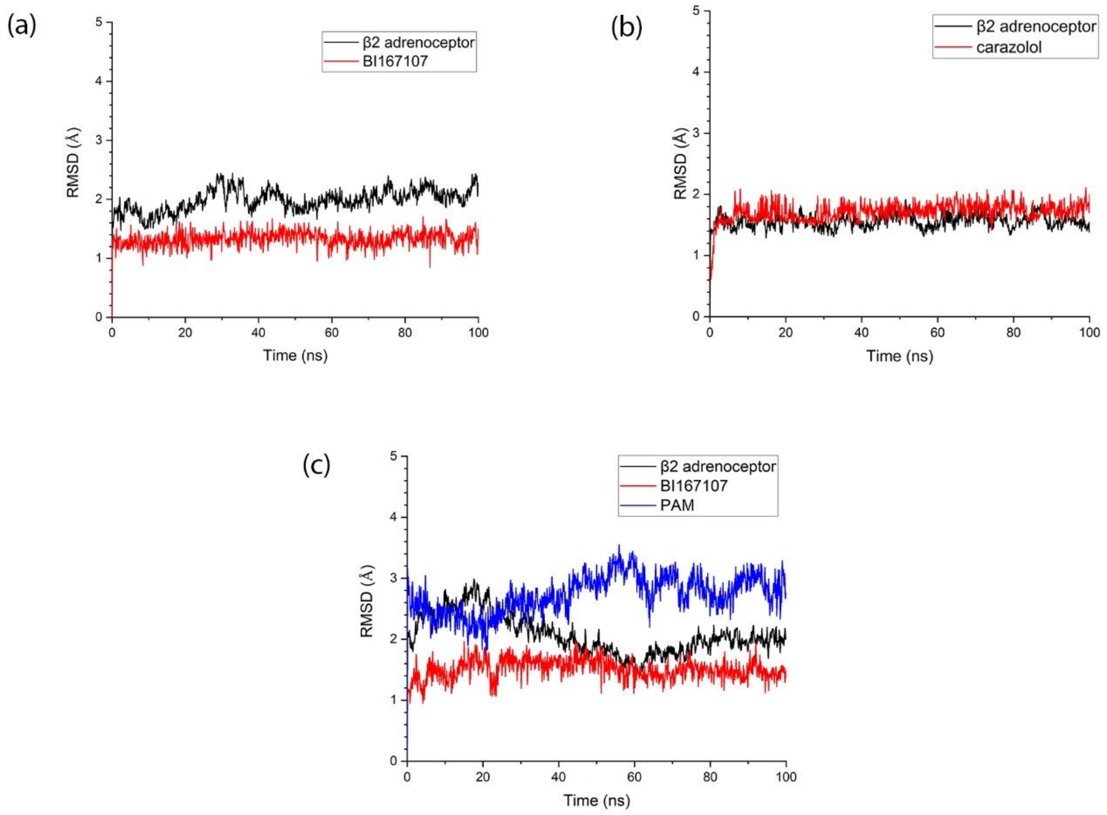

Figure S1. RMSDs graphs of β2 Adrenoceptor system. Black lines represent RMSDs value of β2 Adrenoceptor; Red lines represent RMSDs value of orthosteric ligand; Blue lines represent RMSDs value of allosteric modulator. (a) β2 Adrenoceptor with agonist BI167107; (b) β2 Adrenoceptor with antagonist carazolol; (c) β2 Adrenoceptor with agonist BI167107 and positive allosteric modulator Cmpd-6FA.

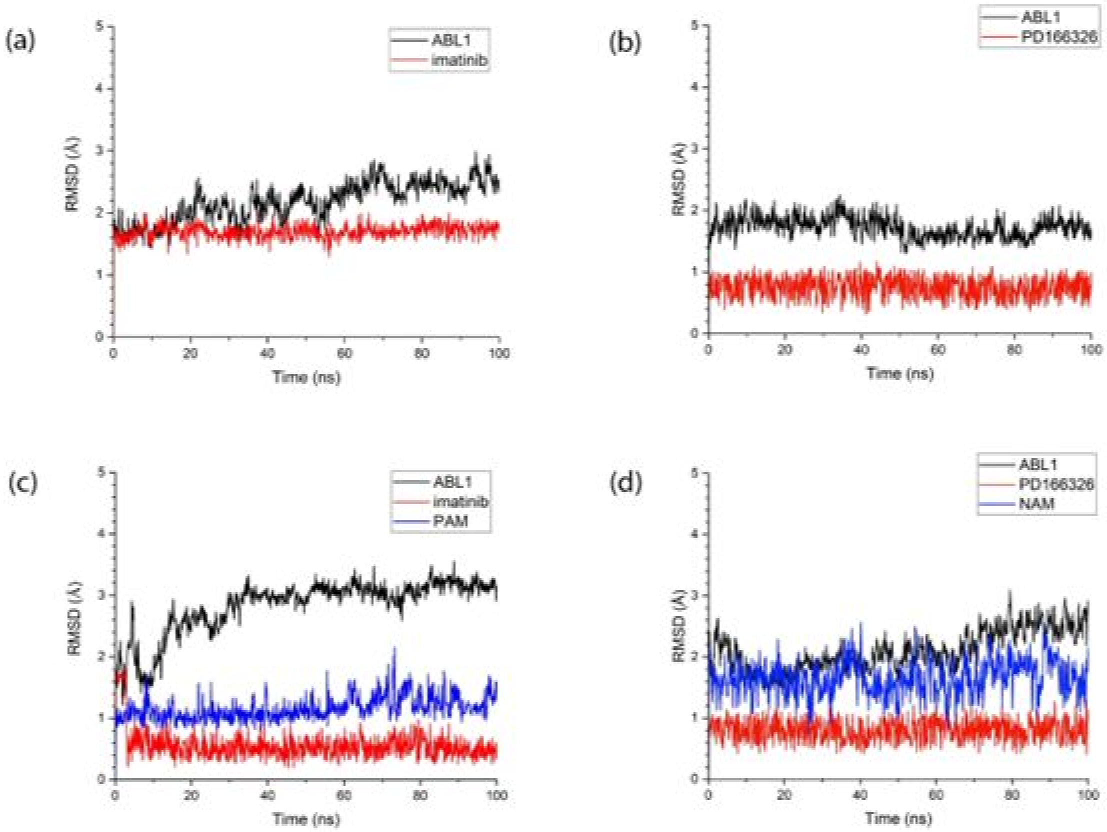

Figure S2. RMSDs graphs of Tyrosine-protein kinase ABL1 system. Black lines represent RMSDs value of ABL1; Red lines represent RMSDs value of orthosteric ligand; Blue lines represent RMSDs value of allosteric modulator. (a) ABL1 with inhibitor imatinib; (b) ABL1 with inhibitor PD166326; (c) ABL1 with inhibitor imatinib and positive allosteric modulator DPH; (d) ABL1 with inhibitor PD166326 and negative allosteric modulator myristic acid.

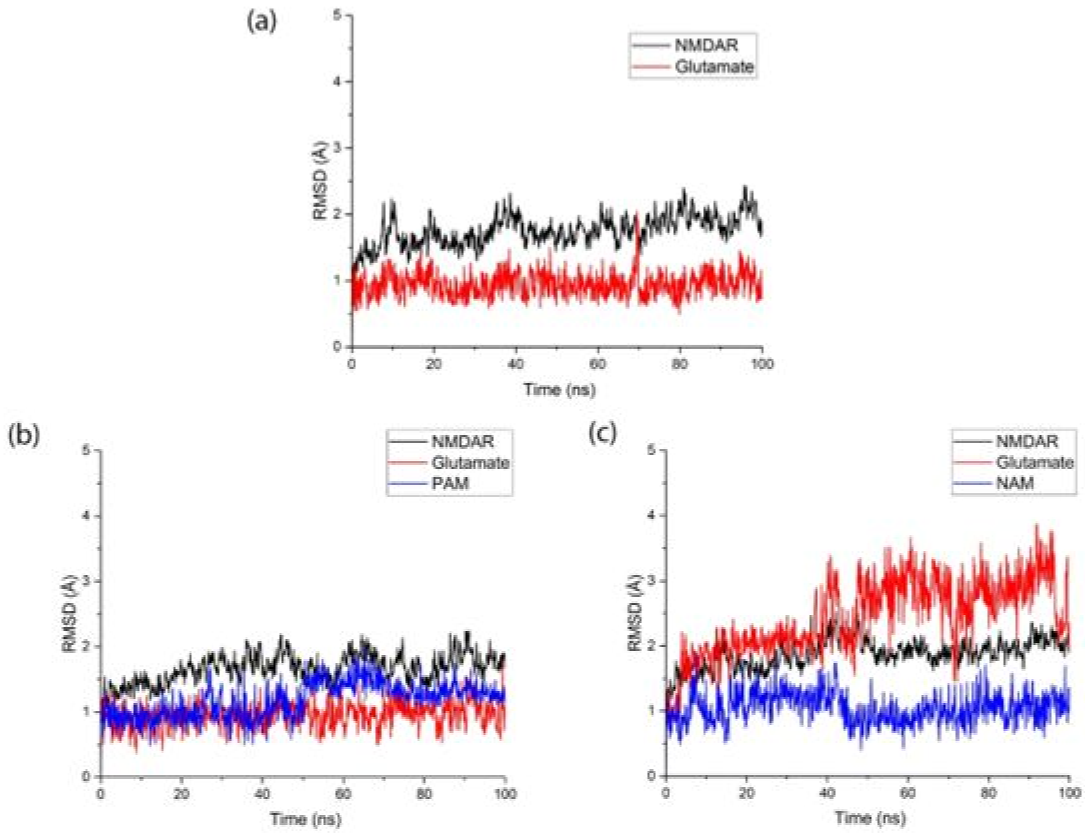

Figure S3. RMSDs graphs of N-methyl-D-aspartate receptor (NMDAR) system. Black lines represent RMSDs value of NMDAR; Red lines represent RMSDs value of orthosteric ligand; Blue lines represent RMSDs value of allosteric modulator. (a) NMDAR with agonist glutamate; (b) NMDAR with agonist glutamate and positive allosteric modulator GNE3419; (c) NMDAR with agonist glutamate and negative allosteric modulator compound 6.

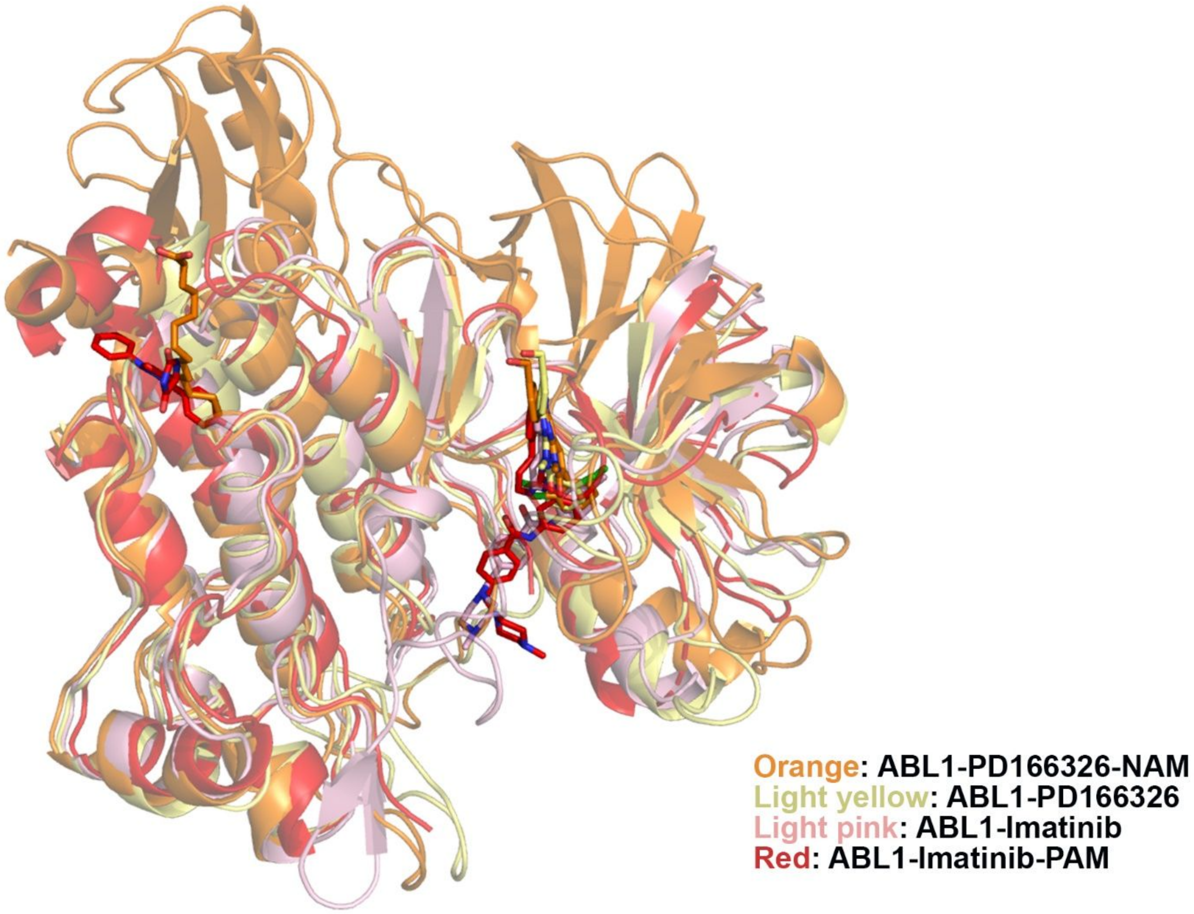

Figure S4. Conformational changes of ABL1 complexes in different states compared by aligning the representative frames of MD simulations. The color orange represents the complex of ABL1-PD166326-NAM (initial MD conformation from PDB ID: 1OPK); the color light yellow represents the complex of ABL1-PD166326 (initial MD conformation from PDB ID: 2G2H); the color light pink represents the complex of ABL1-Imatinib (initial MD conformation from PDB ID: 2HYY); the color red represents the complex of ABL1-Imatinib -PAM (initial MD conformation from PDB ID: 3PYY). The offset distances are marked with green arrows in the figure, and the length units are Å.

Table S1. Ligand-protein binding free energy of all complexes.

Receptor System	PDB ID	Detail (All include receptor)	Binding Free Energy (kcal/mol)
GPCR: β2 adrenoceptor	4LDE	Agonist (BI167107)	-4.03±0.07
	6N48	Agonist (BI167107) + PAM	-1.57±0.19
	2RH1	Antagonist (Carazolol)	-2.25±0.17
	5X7D	Antagonist (Carazolol) + NAM	-15.73±0.54
Enzyme: Tyrosine-protein kinase ABL1	2HYY	Inhibitor (Imatinib)	-25.8±0.28
	3PYY	Inhibitor (Imatinib) + PAM	-6.57±0.26
	2G2H	Inhibitor (PD166326)	-10.58±0.17
	1OPK	Inhibitor (PD166326) + NAM	-1.26±0.15
Ion Channel Receptor: NMDAR	5H8F	Agonist (Glutamic acid, Glycine)	-8.51±0.34
	5H8H	Agonist (Glutamic acid, Glycine) + PAM	-6.44±0.16
	5H8N	Agonist (Glutamic acid, Glycine) + NAM	-13.77±0.55
